# Peripheral venous catheter in cardiology: root cause analysis of an adverse event during patient transport in radiology

**DOI:** 10.1186/2047-2994-4-S1-P210

**Published:** 2015-06-16

**Authors:** D Verjat-Trannoy, A Abdelaziz, V Chauvin, C Hovasse, L Hubas, P Astagneau

**Affiliations:** 1 North France Healthcare Associated Infection Control Centre (CClin Paris-Nord), France; 2 Lariboisière Hospital, Paris, France

## Introduction

The insertion of peripheral venous catheter (PVC) is a basic and trivialized act of nursing with significant associated infection risks. There are few publications about PVC complications compared to those of central lines and the root causes of such events are not well known. In 2014, a survey was conducted to measure the frequency of complications associated with PVC insertions and manipulations.

## Objectives

To select an adverse event (AE) and identify the root causes of its occurrence in order to improve the quality and safety of care of patients with PVC.

## Methods

Daily monitoring of patients and their PVC was carried out by the infection control team during a two months period in a cardiology department. The monitoring data collected were of clinical signs and symptoms as well as of mechanical events. After the selection of an incident, a root cause analysis was performed by Alarm method following the classic plan: chronology of care until the AE occurrence, identification with the nursing team of the immediate causes and of the contributing factors (patient, tasks, individual, team, environment, organization, institution), and action plan.

## Results

A total of 324 PVC were evaluated for 207 hospitalized patients. No major complications were observed during the study. At least one clinical sign or one mechanical problem was identified for 62% of patients and 58% of the PVC. The AE was a partial removal of the PVC associated with obstruction and signs of inflammation occurring during the patient transport for a radiological examination. A root cause analysis was conducted with both the cardiology and the patient transport teams. The main contributing factors identified were factors related to the team and the work environment.

## Conclusion

This epidemiological study led us to a better understanding of the PVC complications and led the care giving teams to a better perception of the risks associated with these devices. In-depth analysis of the causes of this incident resulted in a detailed action plan with a focus on the transportation management of perfused patients. For optimal management of PVC, this approach should be completed by the analysis of other AE identified during the study.

## Disclosure of interest

None declared.

